# Characterization of peach tree crown by using high-resolution images from an unmanned aerial vehicle

**DOI:** 10.1038/s41438-018-0097-z

**Published:** 2018-12-10

**Authors:** Yue Mu, Yuichiro Fujii, Daisuke Takata, Bangyou Zheng, Koji Noshita, Kiyoshi Honda, Seishi Ninomiya, Wei Guo

**Affiliations:** 1 International Field Phenomics Research Laboratory, Institute for Sustainable Agro-ecosystem Services, The University of Tokyo, 1-1-1 Midori-cho, Nishi-Tokyo, Tokyo, 188-0002 Japan; 2 Research Institute for Agriculture, Okayama Prefectural Technology Center for Agriculture, Forestry and Fisheries, 1174-1 Kodaoki, Akaiwa, Okayama 709-0801 Japan; 3 Fukushima University, 1 Kanayagawa, Fukushima, Fukushima 960-1248 Japan; 4 CSIRO Agriculture and Food, Queensland Bioscience Precinct, 306 Carmody Road, St. Lucia, QLD 4067 Australia; 5 JST PRESTO, Kawaguchi Center Building, 4-1-8 Honcho, Kawaguchi, Saitama 332-0012 Japan; 6 Graduate School of Agricultural and Life Sciences, The University of Tokyo, 1-1-1 Yayoi, Bunkyo, Tokyo, 113-8657 Japan; 7 International Digital Earth Applied Science Research Center, Chubu University, 1200 Matsumotocho, Kasugai, Aichi-ken 487-8501 Japan

## Abstract

In orchards, measuring crown characteristics is essential for monitoring the dynamics of tree growth and optimizing farm management. However, it lacks a rapid and reliable method of extracting the features of trees with an irregular crown shape such as trained peach trees. Here, we propose an efficient method of segmenting the individual trees and measuring the crown width and crown projection area (CPA) of peach trees with time-series information, based on gathered images. The images of peach trees were collected by unmanned aerial vehicles in an orchard in Okayama, Japan, and then the digital surface model was generated by using a Structure from Motion (SfM) and Multi-View Stereo (MVS) based software. After individual trees were identified through the use of an adaptive threshold and marker-controlled watershed segmentation in the digital surface model, the crown widths and CPA were calculated, and the accuracy was evaluated against manual delineation and field measurement, respectively. Taking manual delineation of 12 trees as reference, the root-mean-square errors of the proposed method were 0.08 m (*R*^2^ = 0.99) and 0.15 m (*R*^2^ = 0.93) for the two orthogonal crown widths, and 3.87 m^2^ for CPA (*R*^2^ = 0.89), while those taking field measurement of 44 trees as reference were 0.47 m (*R*^2^ = 0.91), 0.51 m (*R*^2^ = 0.74), and 4.96 m^2^ (*R*^2^ = 0.88). The change of growth rate of CPA showed that the peach trees grew faster from May to July than from July to September, with a wide variation in relative growth rates among trees. Not only can this method save labour by replacing field measurement, but also it can allow farmers to monitor the growth of orchard trees dynamically.

## Precision horticulture: Peachy method for monitoring orchard growth

A rapid automated system for monitoring peach tree growth had been developed that could replace laborious field measurements and enable farmers to manage their orchards more effectively.

Knowing a tree’s crown width—the mass of branches and foliage growing outwards from its trunk—enables more effective spraying, machine harvesting, and assessment of trees’ growth. However, because peach trees have irregular shaped crowns, there is no rapid and reliable method for extracting this information. Wei Guo at the University of Tokyo and colleagues used unmanned aerial vehicles to photograph an orchard, then generated a digital surface model which enabled individual trees to be identified, and their crown widths and projection areas to be accurately calculated. This revealed that the trees grew faster during early summer compared to late summer, with wide variation between individual trees.

## Introduction

It is well known that the development of tree canopy affecting both quality and yield of peaches^1^. Especially in Japan, to achieve high economic production, manipulation and management of the tree canopy are essential. As the increasing price of agrochemicals and labour, there is a need for efficient precision management. Precision farming applies the appropriate timing, amount, and location of fertilizer and pesticides to crop management^2^. The preliminary step of precision farming is acquiring as much growth data from the crop as possible^3^, which depends on accurately describing the morphological and structural characteristics of crops. In the pomological aspect, relevant morphological characteristics include crown width, height, area, and volume. Among these, crown width is important for precision spraying^4–6^ and machine harvesting^7^, while crown projection area (CPA) is important for determining tree growth during the growing season^8^.

Several methods are used to measure the crown characteristics. In the field, crown width can be measured by tape or laser rangefinder^9^, and CPA can be approximated as the area of a polygon with points on the crown’s drip-line or estimated from a circle^10^ or ellipse based on the crown spread. Such subjective measurements on a crude scale may introduce large errors^11^, and the precise field measurement of CPA is time consuming^12^. Alternatively, a terrestrial laser scanner can map three-dimensional (3D) plant structure in detail^13^, but the device is costly and accurate crown reconstruction requires high-density laser scan data^14^ which means it consumes more time in data acquisition and processing. The development of computer vision technology has made it possible to build a 3D model of a tree based on the captured photos by camera. This method is not only objective and repeatable compared with field manual measurement, but also more cost effective than using a laser scanner.

According to the platform, cameras mainly include shelf-fixed cameras^8^, handheld cameras^15^, and cameras mounted on unmanned aerial vehicles (UAVs)^7,9,14,16^. Among them, UAVs are rising in popularity for applications in plantations and orchards for its flexibility and relatively larger cover area in data collection^17^. High-resolution images collected from UAVs have been used to determine the height and crown size of olive trees^14,16^, blueberry bushes^7^, Mediterranean riparian forest^18^, and Norway spruce and Scots pine^9^.

In the characterization of trees from UAV images, one of the most important procedures is the extraction of individual trees. A commonly used method for crown segmentation is watershed transformation, which treats the height of trees as terrain and segments individual ‘drainage basins’ by identifying local maxima and nearest minima. Treetops are usually treated as local maxima, but this will cause over-segmentation in structurally complex trees: non-conical shape of crown causes multiple local maxima identified within a single broadleaf crown^9^. Several methods can solve the over-segmentation requiring explicit prior knowledge of the image structure^19^, e.g. multi-scale filtering^20^, marker-controlled watershed^21^, region merging watershed^22^, and watershed method using prior shape^23^. Therefore, finding the simple and effective way to get the location and rough extent of the crown before using watershed segmentation is critical. The commonly used morphological erosion method is sensitive to neighbourhood size and shape, which may eliminate the small trees. Popescu et al.^24^ proposed using small-footprint lidar data to first locate individual trees but assumes that crown diameter is in relation with crown height, which is not suitable for height-controlled fruit trees. In general, correctly isolating individual crowns remains difficult, especially for broadleaf trees with the non-conical shape.

For peach trees trained in an open-centre shape, it is difficult to isolate individual crowns. To the best of our knowledge, the use of UAV imagery of peach trees for crown characterization has not been explored. The primary purpose of this research was to develop an image processing procedure for crown characterization which takes advantage of the crown change of deciduous trees with seasons to extract individual trees. The specific objectives of this study were to (1) propose a new UAV image analysis method for the accurate and efficient characterization of crown width and crown project area of peach trees, (2) evaluate its performance, and (3) use it to analyse the growth of peach trees in 2017 in Okayama, Japan.

## Materials and methods

### Study site description

**Fig. 1:**
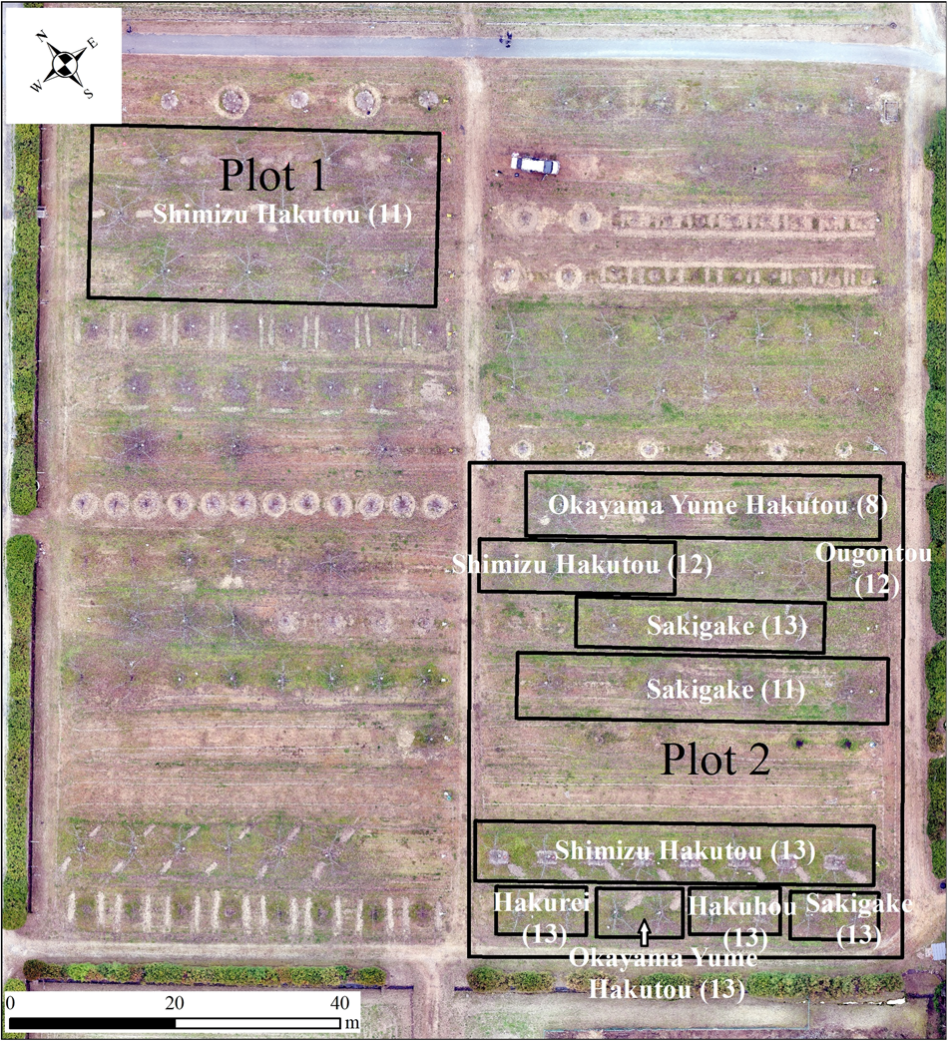
The cultivars and ages by 2018 (in the bracket) of peach trees in the orchard in Okayama, Japan

We tested our method on two plots in a peach orchard (~1.65 ha) in Okayama, Japan. Plot 1 includes twelve 11-year-old peach trees (Shimizu Hakutou) and plot 2 includes 32 peach trees with six different cultivars and nearly 11-years-old (Shimizu Hakutou, Hakuhou, Okayama Yume Hakutou, Hakurei, Ougontou, Sakigake) (Fig. 1). All the peach trees were trained to a modified open-centre shape with two predominant branches oriented approximately perpendicular to the row.

### Data acquisition

We collected three independent datasets: UAV images, manual delineated images, and related field measurement that include crown width and projection area.

#### Collection and pre-processing of UAV images

**Fig. 2:**
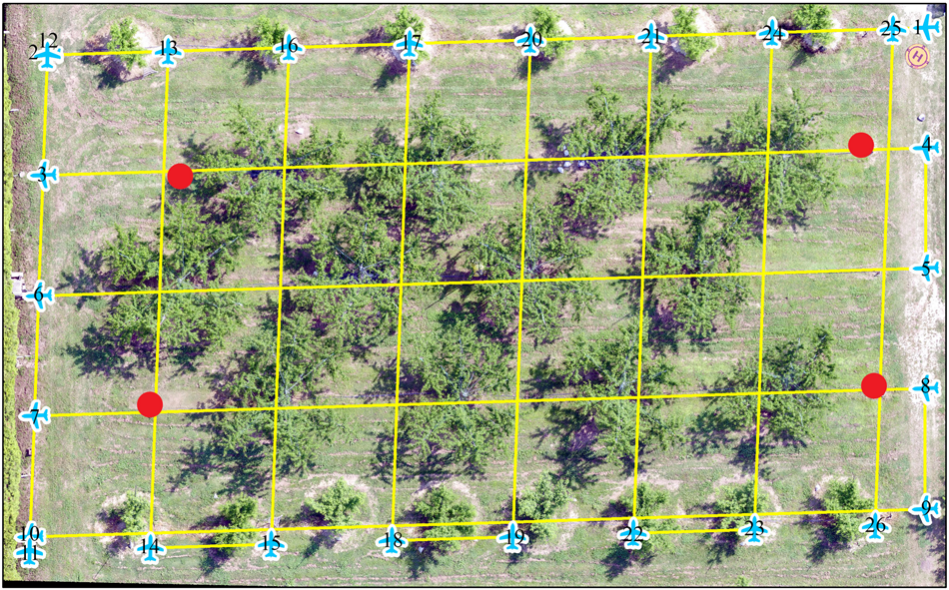
The UAV flight pattern (yellow lines) in plot 1 as an example. It followed a grid at a height of 30 m and a speed of 2.5 m/s, with >80% image overlap. The numbers label the flight order of way point and the red points mark the ground control points

Images for peach trees in plot 1 were collected on five occasions in the orchard from September 2016 to September 2017. Images for peach trees in plot 2 were collected in December 2017 and July 2018 to increase sample size. The trees were photographed with a digital camera mounted on a DJI inspire 1 and inspire 2 UAV (Shenzhen Dajiang Baiwang Technology Co., Ltd, China) with wind speed ≤level 2 according to the Beaufort wind scale. Flight was controlled by Litchi software (VC Technology Ltd, UK), which directed the UAV flying along a double-grid image acquisition plan (Fig. 2) at a height of 30 m and a speed of 2.5 m/s with the camera looking downwards. Overlap of photos to the front and side was >80%. It took about 6 min to cover plot 1 (~0.2 ha) in one UAV flight by DJI inspire 1 and 17 min to cover the whole orchard (~1.65 ha) in one UAV flight by DJI inspire 2. See detailed information about the digital camera and the flight in Table S1. In order to improve the geolocation accuracy, the georeferencing of the point cloud was done using a combination of direct georeferencing and ground control points (GCPs, red points in Fig. 2). The coordinates of the GCPs were measured by Hemisphere RTK differential GNSS devices (Hemisphere GNSS, USA) in plot 1 and Propeller AeroPoints with Propeller correction network (Propeller Aerobotics Pty Ltd, Australia) in the whole orchard. The mean RMSE of the seven flights was 0.24 m.

The raw images collected by UAV were used to generate a 3D point cloud, from which a 2D red-green-blue (RGB) orthomosaic and the digital surface model (DSM) were generated in Pix4Dmapper Pro v. 4.0.25 software (Pix4D Inc., Switzerland) by the Structure from Motion algorithm^25^. Full image scale was set for precisely extracting the key points and the relative camera positions were also taken into account to discard geometrically unrealistic matches. During the generation of DSM, noise filtering was used to correct the altitude of these points with the median altitude of the neighbouring points. The RGB orthomosaic and DSM image from the same point cloud model were exported at the same ground sampling distance. The ground resolution of all RGB and the DSM images was <1 cm/pixel.

#### Manual delineation of trees

The manual delineation dataset was obtained by manually drawing outlines of the 12 crowns from the RGB orthomosaic of September 2016 in Adobe Photoshop software (Adobe Systems Incorporated, USA) and then calculating the two orthogonal crown widths and the CPA based on the drawn crowns and ground resolution in Matlab v. R2017b software (MathWorks Inc., USA).

#### Field measurement

We did the field measurement of peach trees two times. One was in late August 2016 including 12 trees in plot 1 and the other was in early July 2018 including 32 trees in plot 2 as a supplementary experiment. In both the investigations, we measured the crown widths parallel (W_1_) and perpendicular (W_2_) to the row of the trees with a millimetre measuring tapeline. Based on the preliminary experiment, the CPA of each tree was estimated by a local empirical equation:

1 $$CPA_{\mathrm{f}} = 0.65 \times W_{1{\mathrm{f}}} \times W_{2{\mathrm{f}}}$$

where W_1f_ and W_2f_ represent the crown widths parallel and perpendicular to the row of the trees, respectively.

### Characterization of peach trees

The geometric characteristics of crowns were calculated from the DSM in Matlab v. R2017b software (MathWorks Inc., USA). The crown geometry is derived using two kinds of DSM (bare-branch DSM and foliated DSM) by image analysis techniques in the following five steps (source codes and sample data are available at our surpport page: https://github.com/UTokyo-FieldPhenomics-Lab/Characterization-of-peach-tree-crown):

1. For both kinds, the crowns were first identified from the DSM at an adaptive threshold^26^, and the region of the target field was extracted with a fixed polygon mask (red lines, Fig. 3a, g). Then the DSM of the target peach trees was extracted (Fig. 3h).

**Fig. 3:**
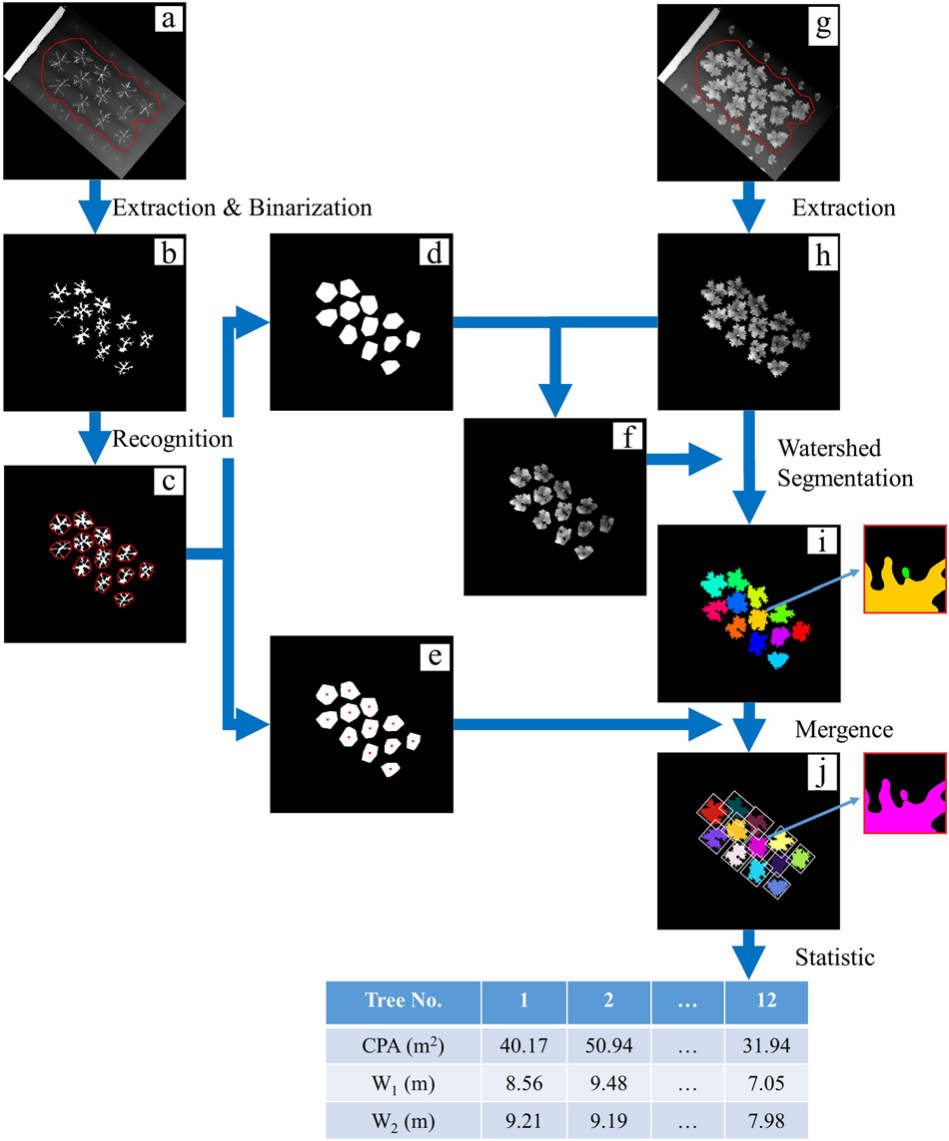
Workflow of the proposed method. **a** Original DSM of trees in winter (red line is the boundary of the polygon mask). **b** Crown scaffolding extracted by adaptive threshold and morphology operation ‘Closing’. **c** Convex polygons (red line is the boundary). **d** Convex masks. **e** Centroids of the convex polygon (red points). **f** Marker used in watershed segmentation, which is the intersection of (**d**) and (**h**). **g** Original DSM of trees in growing season. **h** Extracted DSM of crowns by adaptive threshold algorithm. **i** Segmented trees by marker-controlled watershed algorithm. **j** Individual trees after merging some small parts (white boxes are bounding boxes)
2. For the bare-branch DSM (generated from photos taken on February 2017; Fig. 3a), the mathematical morphology operation ‘Closing’ (structural element: diamond, radius: 50 pixels (February 2017) and 80 pixels (December 2017)) was used to merge the branches of each tree as the scaffolding (Fig. 3b). Then the convex boundary of each tree was delimited (red lines, Fig. 3c).
3. For the foliated DSM (generated from photos taken in the growing season; Fig. 3g), using the intersections of the convex mask (Fig. 3d) and the extracted DSM (Fig. 3h) as a marker (Fig. 3f), watershed transformation^27^ was conducted on the extracted DSM, and then individual crowns were extracted (Fig. 3i).
4. Small isolated parts of images (enlarged view in Fig. 3i) were merged into the closest crown (determined from the distances to the centroids of each convex) (Fig. 3j) and then labelled with a unique ID.
5. The CPA of each tree was calculated as the product of the number of pixels in each crown and the square of ground resolution (m^2^/pixel). The two orthogonal crown widths (W_1_ and W_2_) corresponded to the width and length of the bounding box parallel to the row (Fig. 3j) and were converted to metres (m) by multiplying with the ground resolution (m/pixel).

### Evaluation and comparison of the accuracy of the method

To comprehensively analyse the error of the proposed method, we evaluated the accuracy of the estimated crown widths and projection area against two referential datasets. The manual delineation dataset was used from the perspective of image analysis and field measurement dataset was used from the perspective of horticulture research. The accuracy of the proposed method was evaluated by calculating the square of the correlation coefficient (*R*^2^) and the root-mean-square errors (RMSE) and relative (R)-RMSE against the referential data in Matlab R2017b as:

2 $$RMSE = \sqrt {\frac{{\mathop {\sum}\nolimits_{i = 1}^n {Err_i^2} }}{n}} = \sqrt {\frac{{\mathop {\sum}\nolimits_{i = 1}^n {(v_i - v_{ri})^2} }}{n}}$$

3 $$R{\mathrm{ - }}RMSE = \frac{{RMSE}}{{\frac{{\mathop {\sum}\nolimits_{i = 1}^n {v_{ri}} }}{n}}}$$

where Err_*i*_ is a measurement error of the *i*^th^ tree, *v*_*i*_ is a measurement value of the *i*^th^ tree, *v*_*ri*_ is a referential value of the *i*^th^ tree, and *n* is the number of trees.

### Calculation of the growth rate

CPA can be used to determine tree growth during the growing season^8^, and growth rate was proved to be helpful to understand the vigour of a tree^28^. The growth rate (GR) of CPA was calculated as Eq. (4), and the relative growth rate (RGR) was calculated as Eq. (5):

4 $$GR = \frac{{CPA_t - CPA_{t - {\mathrm{\Delta }}t}}}{{{\mathrm{\Delta }}t}}$$

5 $$RGR = \frac{{CPA_{\mathrm{t}} - CPA_{t - {\mathrm{\Delta }}t}}}{{CPA_{t - {\mathrm{\Delta }}t} \times {\mathrm{\Delta }}t}}$$

where Δ*t* is the interval of days.

## Results

### Peach trees extracted by the proposed method

**Fig. 4:**
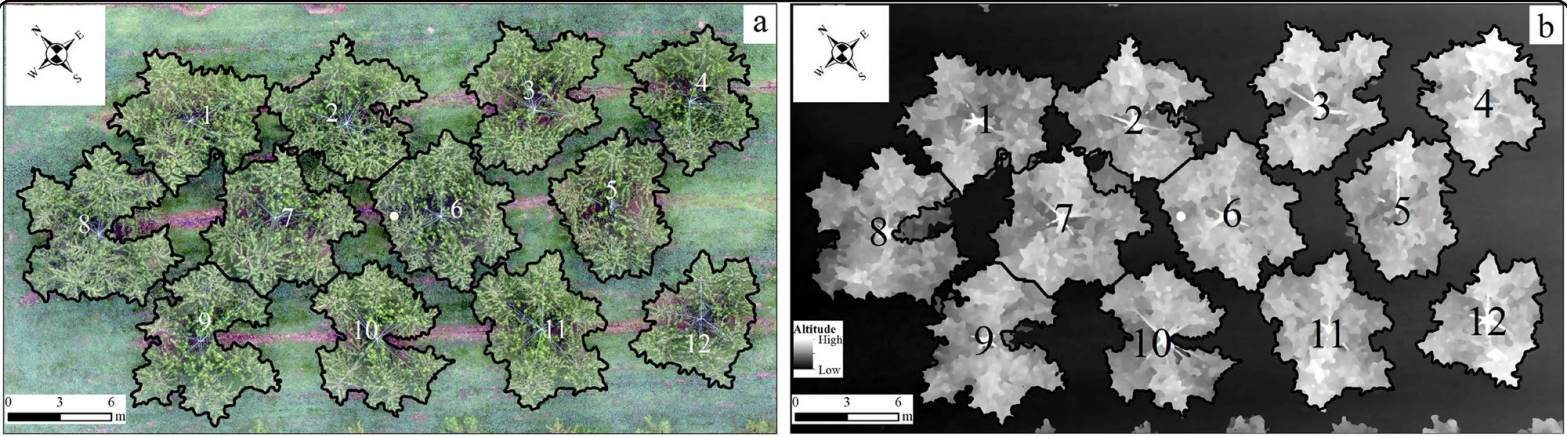
Individual crowns (September 2016) extracted by the proposed method. a and b shows the outlines of individual crowns in RGB orthomosaic and DSM

**Fig. 5:**
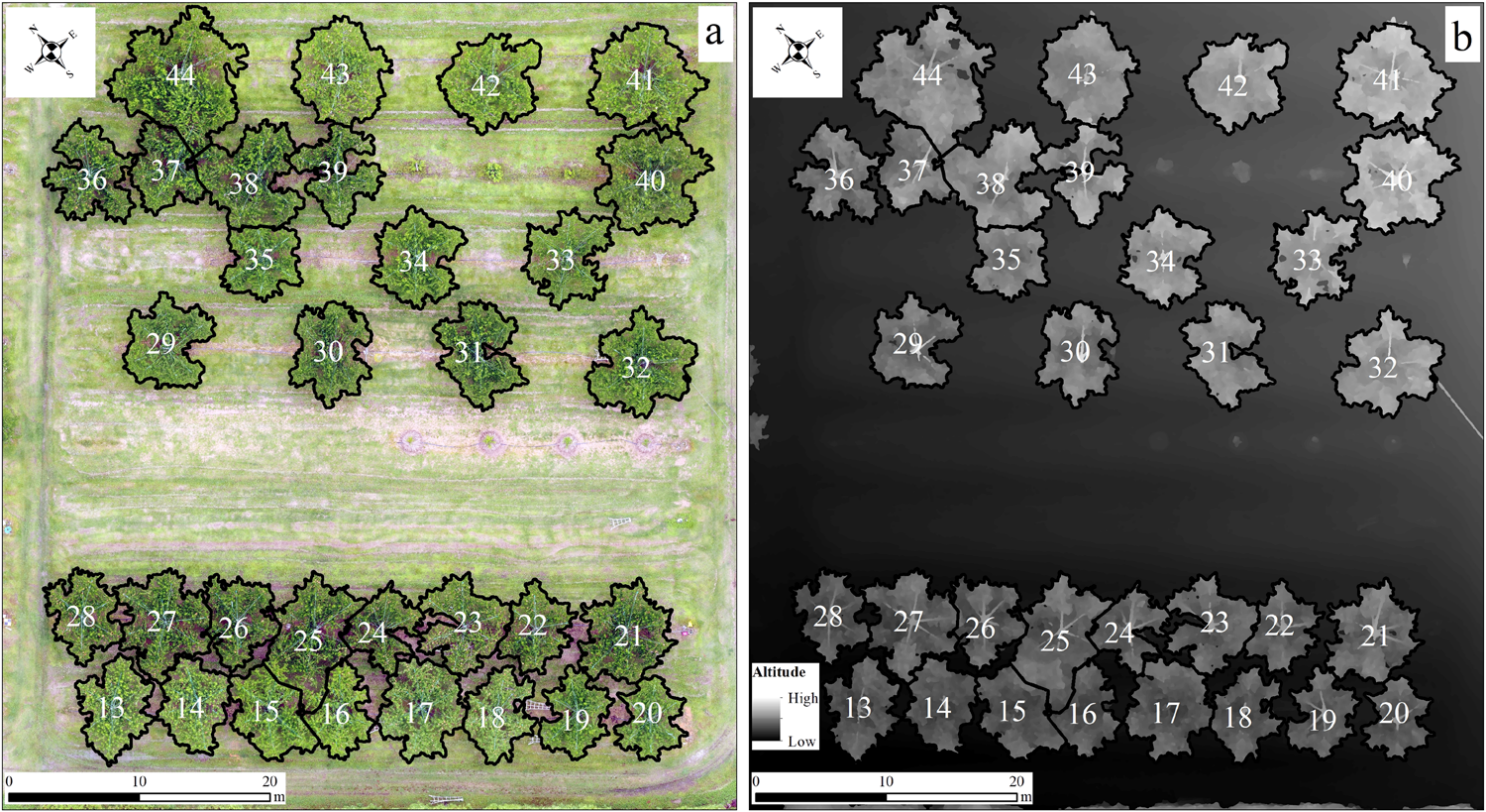
Individual crowns (July 2018) extracted by the proposed method. a and b shows the outlines of individual crowns in RGB orthomosaic and DSM

By using the proposed method, peach trees in plots 1 and 2 were extracted and characterized. In plot 1, almost all the trees were segmented well (Fig. 4a) but the crown of tree Nos. 2, 7, and 8 lost a little bit. The loss of crown was caused by the lower sensitivity when using adaptive threshold algorithm as some parts of the crown was not high enough (Fig. 4b). In plot 2, most crowns were separated well except that of tree Nos. 44, 37, 38, 25, 15, and 16 (Fig. 5a). It was due to the crowns of tree Nos. 25, 15, and 16 were too close that the overlap of branches pushed up the height on the boundary (Fig. 5b), as well as tree Nos. 44, 37, and 38.

### Evaluation of the accuracy of the proposed method with manual delineation

To evaluate the proposed method from the perspective of image analysis, taking crowns delineated (‘d’) manually from the RGB orthomosaic as referential data, the accuracy of the crown widths and CPA determined by the proposed method (‘p’) from a DSM was evaluated according to the calculated *R*^2^, RMSE, and R-RMSE.

**Fig. 6:**
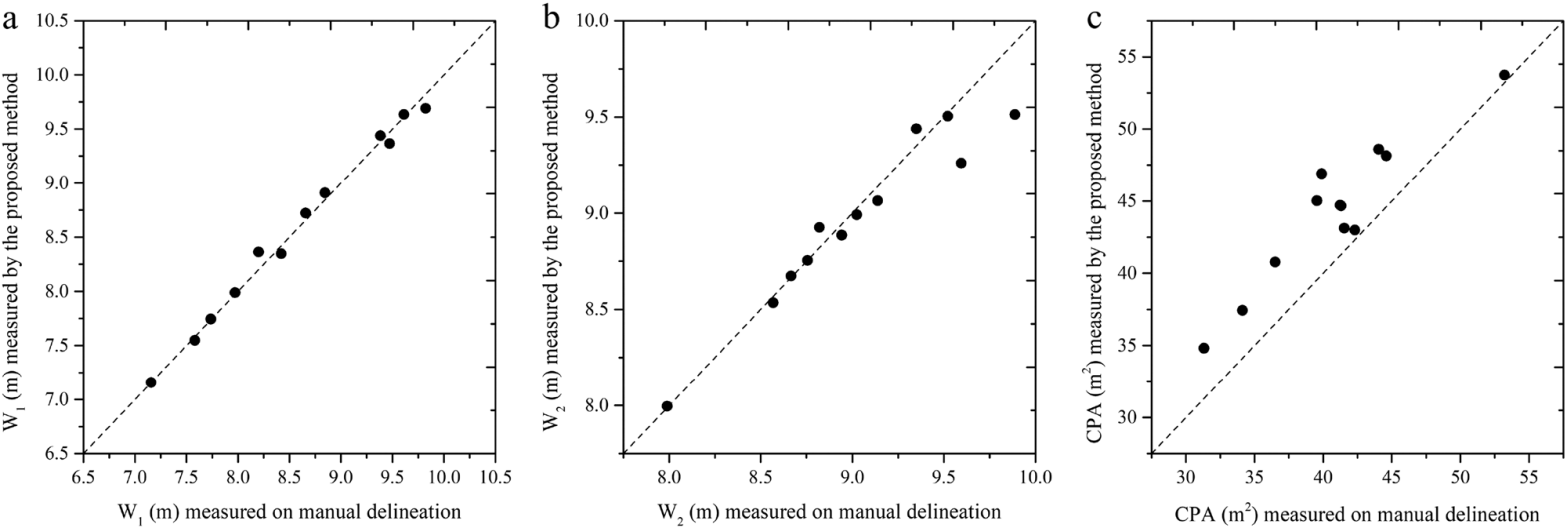
Evaluation of the accuracy of crown width and crown projection area (CPA) against manual delineation. **a**, **b**, and **c** shows the accuracy of W_1_, W_2_ and CPA, respectively

**Fig. 7:**
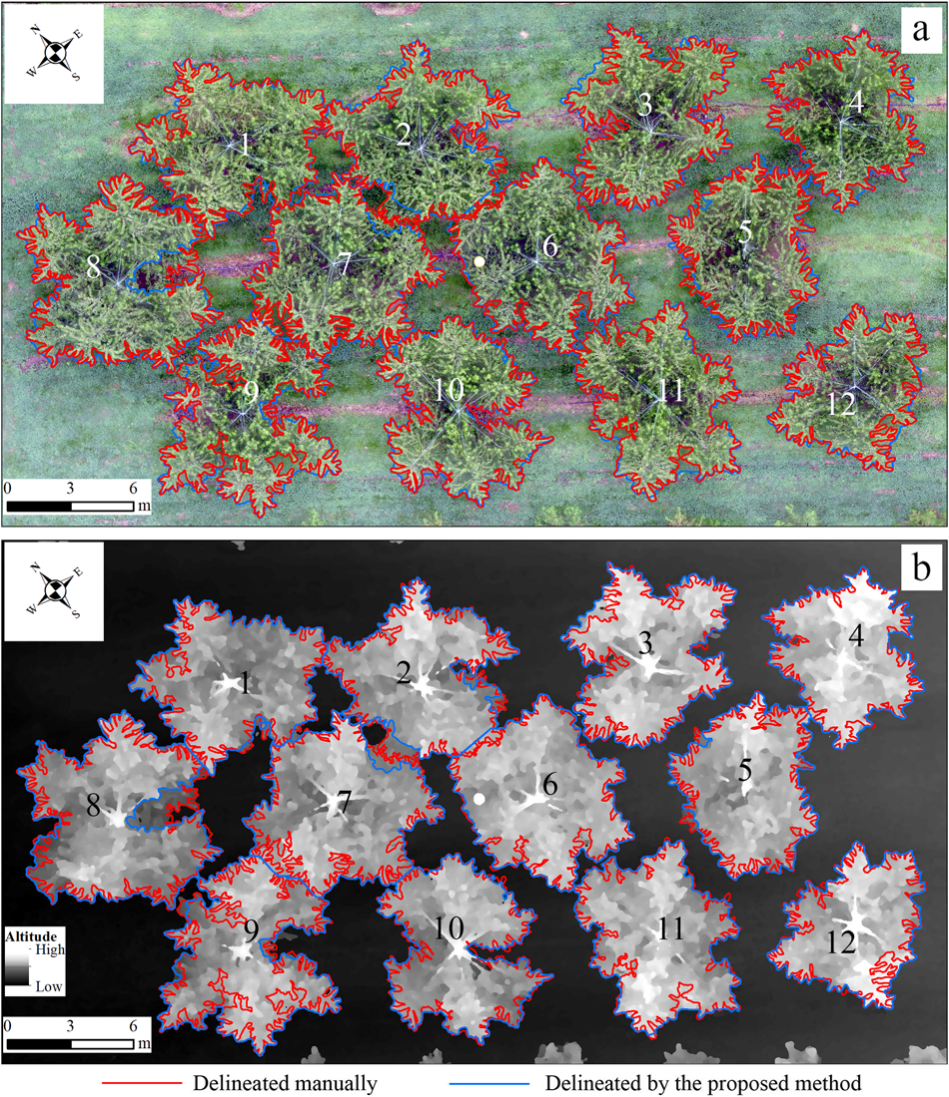
Comparison of individual crowns delineated by the proposed method (blue) and manually (red). a and b shows the outlines of individual trees photoed on September 2016 in RGB orthomosaic and digital surface model

The accuracy of W_1p-d_ and W_2p-d_ was high as shown in Table 1. The *R*^2^ of CPA_p-d_ was also high, but the R-RMSE of CPA_p-d_ was relatively large compared with W_1p-d_ and W_2p-d_, and all CPA_p_ were larger than the reference area (Fig. 6c). It may be caused by the coarser resolution of DSM compared with the RGB orthomosaic, by comparing the manual delineation outline in Fig. 7a, b. From a viewpoint of image analysis, the accuracy of the proposed method was acceptable.

**Table 1:** The square of the correlation coefficient (*R*^2^), root-mean-square errors (RMSE), and relative (R)-RMSE of the proposed method with manual delineation measurement as reference

Feature	*R* ^2^	RMSE	R-RMSE
W_1p-d_	0.99**	0.08 m	0.92%
W_2p-d_	0.93**	0.15 m	1.71%
CPA_p-d_	0.89**	3.87 m^2^	9.49%

**p-value < 0.001, *n* = 12

### Evaluation of the accuracy of the proposed method with field measurement

**Fig. 8:**
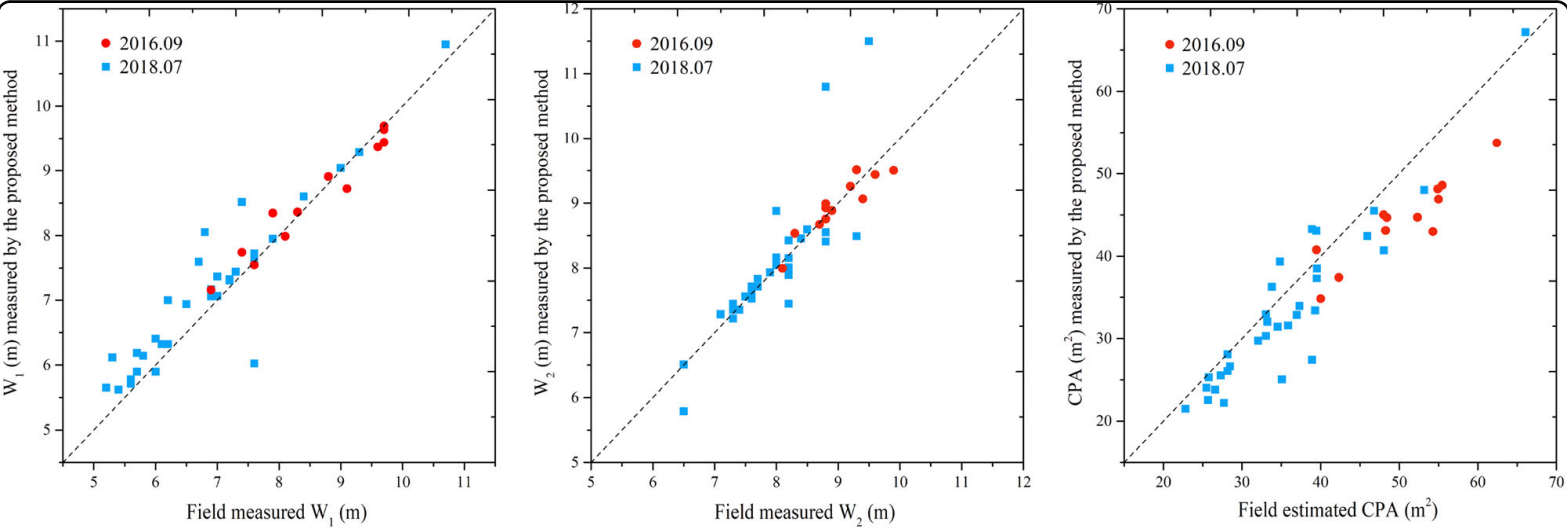
Evaluation of the accuracy of crown width and crown projection area (CPA) against field measurement. **a**, **b**, and **c** shows the accuracy of W_1_, W_2_ and CPA, respectively. The red points represent trees measured on September 2016 and the blue points represent trees measured on July 2018

Considering crowns may overlap, from the viewpoint of horticulture research, the accuracy of the proposed method was also evaluated by the field (‘f’) measurement. From Fig. 8a, b, it is shown most of the crown widths determined by the proposed method were close to those measured in the field, except some crowns not well segmented as analysed in the ‘Peach trees extracted by the proposed method’ section. The *R*^2^ of W_2p-f_ was lower than W_1p-f_ as shown in Table 1, which was caused by the large error of those crowns, e.g. tree Nos. 25, 15, and 16 (Fig. 5).

**Fig. 9:**
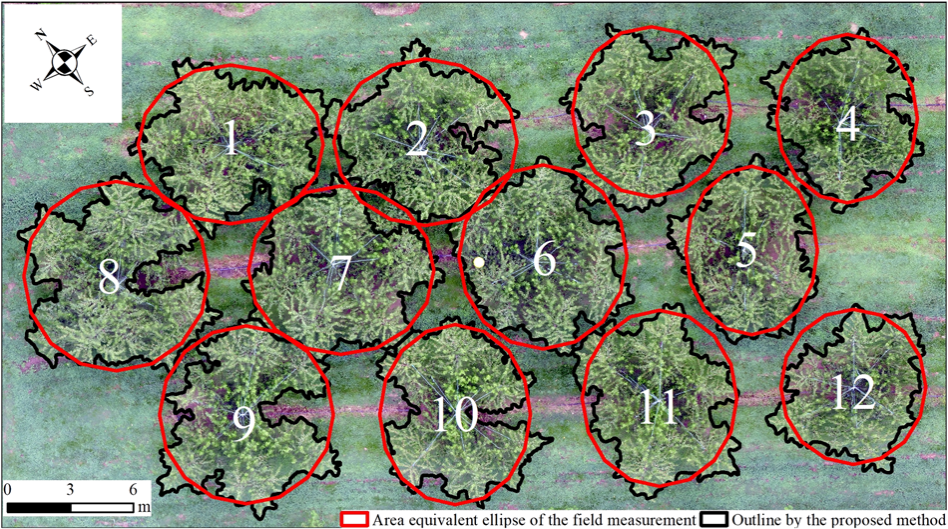
Comparison of the field estimated area and the determined area by the proposed method. The ellipse (in red) was drawn with the same area of field estimation, by taking the W_1_ and W_2_ as the major and minor axis, which shrank with the same scale

Compared with field estimation, the CPA_p_ tends to be underestimation with the CPA_f_ increasing. The *R*^2^ of CPA_p-f_ was also high, but the R-RMSE of CPA_p-f_ was relatively large as shown in Table 2. The error may come from three aspects, (1) the propagation of error in crown width determination, (2) the underestimation of CPA due to the overlap of the crown on the boundary, (3) the error in estimating the area by using the empirical equation, Eq. (1). Taking the crowns in plot 1 as an example, Fig. 9 demonstrated the discrepancy in area calculated by Eq. (1) and the proposed method. Tree No. 5 has the closest value with field estimation with a difference of 1.31 m^2^, while that for tree No. 10 was 5.15 m^2^. It seems if the shape of the crown was non-ellipse-like, the error would be larger than ellipse-like.

**Table 2:** The square of the correlation coefficient (*R*^2^), root-mean-square errors (RMSE), and relative (R)-RMSE of the proposed method with field measurement as reference

Feature	*R* ^2^	RMSE	R-RMSE
W_1p-f_	0.91**	0.47 m	6.38%
W_2p-f_	0.74**	0.51 m	6.25%
CPA_p-f_	0.88**	4.96 m^2^	12.54%

**p-value < 0.001, *n* = 44

### Application in growth analysis of peach tree crowns

CPA can be used to determine tree growth during the growing season^8^, and vegetative growth rate is closely related with vigour^28^. We calculated the CPA of the 12 trees in plot 1 from May to September 2017.

**Fig. 10:**
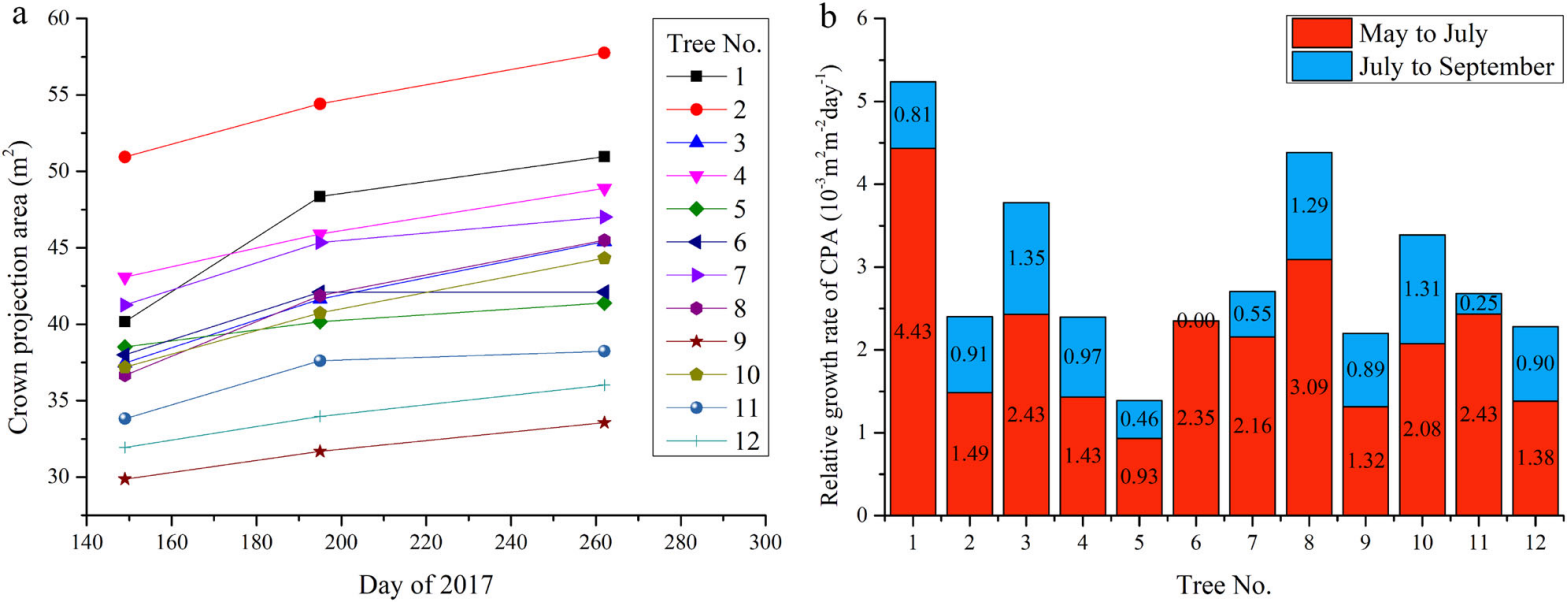
Changes in crown projection area (CPA) and its relative growth rate of  trees in plot 1 during the growing season in 2017. **a** shows the changes in CPA and **b** shows the changes in relative growth rate of CPA

During the growing season, CPA increased consistently (Fig. 10a) by an average of 6.02 m^2^. From May to July, it increased at a mean GR of 8.14 × 10^−2^ m^2^ day^−1^. From July to September, it increased at a mean GR of 3.40 × 10^−2^ m^2^ day^−1^. The GRs of CPA from May to July were larger than that from July to September for all the trees (Fig. 10a). Thus, the trees grew faster from May to July than from July to September.

The RGRs of CPA differed among trees (Fig. 10b). From May to July, the CPA of tree No. 1 increased most (4.43 × 10^−3^ m^−2^ day^−1^) and that of tree No. 5 increased least (0.93 × 10^−3^ m^−2^ day^−1^). From July to September, the CPA of tree No. 3 increased most (1.35 × 10^−3^ m^−2^ day^−1^) and tree No. 6 almost not increased. Overall, tree No. 5 had the lowest mean RGR of CPA (0.69 × 10^−3^ m^−2^ day^−1^).

## Discussion

### Accuracy comparison with other researches

We demonstrated a UAV image analysis method for accurate and efficient determination of crown width and CPA of peach trees and evaluated its accuracy against field measurements. In comparison with other researches as shown in Table 3, the proposed method had relatively high accuracy for both crown width and CPA (Table 3). However, the high ground resolution (<1 cm/pixel) may more or less contribute to the high accuracy.

**Table 3:** The accuracy comparison with other researches taking field measurement as reference data

Feature	*R* ^2^	R-RMSE (%)	Ground resolution (cm/pixel)	Author
Crown width	0.63	14.29	5	Panagiotidis et al.^9^
	0.85	18.56		
	0.58	18.83	8	Díaz-Varela et al.^14^
	0.92		<1^a^	Patrick and Li^7^
	0.91	6.38	<1	Proposed method
	0.74	6.28		
Crown projection area	0.7^b^		<1^a^	Patrick and Li^7^
	0.88	12.54	<1	Proposed method

^a^ Ground resolution was estimated according to the flight height and Figure 14 in Patrick and Li^7^

^b^ The correlation was between the crown projection area field manually measured and determined by bush boundary of the point cloud

### Comparison with field measurement in work efficiency

The UAV imaging method and the data processing workflow that we developed are applicable to practical orchard management. It took about 6 min to photograph 12 trees, while it took about 30 min to measure the crown widths by using tapeline in the field. Using UAV for data collection largely saved time consumed in the field. In data processing, it took about 3 h for DSM generation using ~150 photos followed by about 50 s to determine the canopy characters on a PC with 64 GB of RAM, Intel i7-5930K CPU and 64-bit Windows 10 operating system. All the processing steps could be done automatically, and the measurements are repeatable, which is considered more reliable and efficient than traditional field measurement.

### Error analysis

From the viewpoint of image analysis, according to accuracy evaluation with manual delineation, the error of CPA > W_2_ > W_1_. The error of W_2_ was larger than W_1_ mainly due to crown segmentation error. There were more touching branches at the dominant branch direction, which not only increased the segmentation task by using the watershed algorithm, but also may push up the altitude by overlapping (Fig. 5b). The error of CPA mainly due to crown segmentation error by using adaptive threshold and watershed algorithm, and the coarser resolution of DSM than RGB orthomosaic. The less sensitivity of some lower branch made them failed to be extracted. The representation of the convex hull in winter to the actual shape of the crown may also influence the segmentation while using watershed algorithm. In addition, the systematic overestimation of CPA (Fig. 6c) was likely caused by the noise filter during the DSM generation process: the same crown looks bloated in the DSM image than in the RGB orthomosaic. During the generation of the DSM, the points on the border of ground and crown may be interpreted as noisy and erroneous points by Pix4Dmapper because of huge change in altitude, and their altitude were corrected with the median altitude of neighbouring points. Thus made the height of points inside the crown become smooth. This kind of smooth effect made the crown looks bloated.

From the viewpoint of horticulture research, the overlapping of crowns may lead underestimation of CPA (Fig. 9). In addition, field measurement of crown widths, and more so estimation of crown project area, has inherent error^14^. In the measurement of crown width, it may bring error in confirming the farthermost point of the crown, especially for at the parallel to row direction, as people not easy to stand near the farthermost points at the same time, e.g. tree No. 11 (Fig. 9). Because the crown project area of peach tree is estimated by a local empirical equation, which calculated the ellipse area with coefficient adjustment, the shape of the crown being non-ellipse-like may get larger estimation error than ellipse-like.

### Limitations of the proposed method

Though the proposed method was shown to be accurate and efficient, there are some limitations. One limitation is it only measured the crown on the top view, which means error will increase if there are too much overlaps between each tree. Another limitation of using the proposed method is that the scaffold branches themselves must be separated. Although this is applicable to most fruit trees as the requirement of orchard management, it is difficult to separate trees in the forest of high density. The method was tested in only one flat orchard of the same training strategy. More orchards with different training strategies need to be tested in the future.

### Prospective

Growers pay particular attention to vegetative growth, which competes with fruit growth and influences potential yield^28,29^. And by setting varied fertilizer management zones using spatio-temporal analysis of field characteristic and previous yields maps, it can achieve high environmental and economic benefits^30^. The proposed method allowed us to calculate the GR and RGR of each tree (‘Evaluation of the accuracy of the proposed method with field measurement’ section), which could help not only make a reasonable pruning or growth regulation plan, but also a varied fertilizer management.

## Conclusions

This study confirms morphological traits, such as crown size and area, can be extracted from height information of peach trees generated by using consumer-grade UAV imagery and computer vision techniques. The results revealed that the crown size and area derived by our method were close to the manually delineation and field measured, and it could replace field measurement to achieve significant labour saving. The change of CPA showed the growth rate of peach tree was larger from May to July than from July to September and have a large variety on different trees. Furthermore, the findings of this study lead us to believe that this technology would allow for the convenient surveillance of orchard trees to observe growing trends and could therefore provide guidance in fruit tree management.

## Electronic supplementary material

Detailed information about the UAV flight
