# Supplementary material for: Characterization of peach tree crown by using high-resolution images from an unmanned aerial vehicle
Source: Hortic Res. 2018 Dec 10;5:74. doi: 10.1038/s41438-018-0097-z (PMC6286954; doi:10.1038/s41438-018-0097-z)
Supplement: Supplementary file 1 — Detailed information about the UAV flight [file 41438_2018_97_MOESM1_ESM.docx]

***Horticulture Research* Supporting Information**

Article title: **Characterization of peach tree crown by using high-resolution images from an unmanned aerial vehicle**

**Table S1** Detailed information of UAV flight.

| Date | Drone | Camera | Effective  (megapixels) | Focal length | Number of participated  photos ^a^ | Wind speed ^b^ (mph) |
| --- | --- | --- | --- | --- | --- | --- |
| 2016.09.06 (plot 1) | DJI inspire 1 | ZENMUSE X5 | 16 | 15 mm | 104 | 3.3 |
| 2017.02.08 (plot 1) | DJI inspire 1 | ZENMUSE X5 | 16 | 15 mm | 166 | 1.1 |
| 2017.05.29 (plot 1) | DJI inspire 1 | ZENMUSE X5 | 16 | 15 mm | 131 | 2.5 |
| 2017.07.14 (plot 1) | DJI inspire 2 | ZENMUSE X5S | 20.8 | 15 mm | 123 | 1.0 |
| 2017.09.19 (plot 1) | DJI inspire 1 | ZENMUSE X5 | 16 | 15 mm | 236 ^c^ | 1.5 |
| 2017.12.15 (whole orchard) | DJI inspire 1 | ZENMUSE X5 | 16 | 15 mm | 381 | 1.5 |
| 2018.07.04 (whole orchard) | DJI inspire 2 | ZENMUSE X4S | 20 | 8.8 mm | 432 | 6.3 |

^a^ Number of participated photos indicate the number of photos used for 3D reconstruction in Pix4D.

^b^ Wind speed was acquired by Pix4D report and flight logs in <https://airdata.com/>.

^c^ The photos covered the plot 1 two times.
